# Images of time: temporal aspects of auditory and movement imagination

**DOI:** 10.3389/fpsyg.2014.00877

**Published:** 2014-08-12

**Authors:** Rebecca S. Schaefer

**Affiliations:** Department for Psychological and Brain Sciences, SAGE Center for the Study of the Mind, University of California, Santa BarbaraSanta Barbara, CA, USA

**Keywords:** auditory imagery, motor imagery, temporal processing, expertise

Research on mental imagery has shown that when we imagine something, the related neural processes overlap with those related to actually perceiving or performing that same percept or action (Kosslyn et al., 2001). Although visual imagery has long been the dominant modality for the investigation of sensory imagery, involvement of modality-specific brain regions (i.e., visual areas being implicated in visual imagery, and so on) has now also been reported for auditory, olfactory and tactile imagery (Halpern, 2001; Plailly et al., 2012; Schmidt et al., 2014). Movement or action imagery has informed theories of action representation (Jeannerod and Decety, 1995) and has recently gained interest in the context of mental practice for expert skill acquisition such as sports or surgery (Cocks et al., 2014) and movement rehabilitation (Malouin and Richards, 2010). Increasingly, the neural underpinnings of imagery have become clearer, and both modality-specific and modality-unspecific neural activations related to imagery have been found (Daselaar et al., 2010; Zvyagintsev et al., 2013). However, the growing body of neuroimaging literature on imagination has yet to include an account of temporal imagery. Although the reproduction of time intervals has been a research topic of interest, the contribution of temporal imagery—namely internal timekeeping or creating the temporal aspects of imagery in other modalities—is largely unexplored. As such it is unclear whether, similar to other perceptual modalities, imagery for time shares cerebral substrate with brain networks involved in time perception or regularity detection. Specifically for actions or sounds, the temporal structure of the imagined stimulus or action is crucial in conjuring a faithful image, suggesting that in scientific findings of auditory and motor imagery, temporal imagery is included. The temporal patterns we create internally arguably lie at the basis of any self-paced movement, be it a fast sprint, a musical performance, or an easy walk in the park. Here, I argue that the shared components between movement and auditory (specifically music) imagery may offer a window into timing and temporal skills, which may carry cognitive importance beyond movement or auditory functions.

Whereas the reciprocal influences of rhythmic sound and movement have been shown, with rhythmic movement supporting auditory acuity (Su and Pöppel, 2012) and rhythmic sound supporting movement efficiency (Bood et al., 2013), few studies have looked at the extent to which imagery can functionally replace perception or action and show similar interactions, which may point to shared processing in their temporal structure. Music imagination was reported to affect finger tapping similarly to perceived music (Repp, 2001) and has been used to support movement in clinical settings (Satoh and Kuzuhara, 2008). Anecdotal observation from music imagery experiments (such as described in Schaefer et al., 2011b), suggests that tapping a finger along to music imagery supported accurate imagery timing, and auditory cues are reported to increase motor imagery vividness (Heremans et al., 2009). Furthermore, motor timing skills and anticipatory music imagery ability appear to be related (Pecenka and Keller, 2009; Keller and Appel, 2010). These findings suggest that the links between motor and music processing may extend into imagined stimuli or actions, and the contribution of the current piece is to bring together findings from temporal processing, music and motor research, and to assert that firstly, the commonalities between music and motor imagery may reveal more about internally driven timing mechanisms, and secondly, one might speculate that these internal timing patterns are related to expertise, and as such may be seen in the context of findings on temporal skills and cognitive abilities (Madison et al., 2009; Holm et al., 2011).

## Imagery for temporal patterns

Auditory patterns and movement sequences share a strong dependence on temporal structure. Specifically in music and speech sounds, a fine-grained temporal ordering of acoustic features forms a meaningful percept, and precise perceptual skills are required to decode or extract information from these signals. Similarly, skilled movement sequences are intricately ordered, and often need to be concatenated through extensive practice. When sounds or complex movements are imagined, their temporal structure crucially needs to be integrated in the image, creating an imagined temporal pattern. The direction of time in imagined sounds or movement sequences is non-reversible, although single elements can be imagined in reverse; for instance, you may imagine single movement elements in reverse, or a construct working memory task that makes use of sensory imagery through the mental reversal of melodies, see Zatorre et al. (2009), although this changes the identity of the sound or movement, and likely requires increased imagery ability and domain-specific expertise.

The discourse on the temporal structure of imagery is most developed for the motor domain, where researchers aim to understand and increase the efficacy of the mental practice of skilled movement (for reviews, see Guillot et al., 2012; Smith and Wakefield, 2013). Although the timing of an imagined action will not necessarily convey information about the content or vividness of the imagery, the assumption is that it at least gives an indication of whether the temporal structure of the imagined action is similar to the actual action. Among the factors that can affect the timing of motor imagery are the type, complexity and duration of the imagined action and the age, expertise level and imagery strategy of the subject (Guillot et al., 2012). Although there is still little evidence to suggest that accurately timed mental practice actually improves motor learning, the timing of movement imagination has been shown to impact the timing of subsequent motor performance (Debarnot et al., 2011). Again, changing the speed of a complex movement changes its nature (although much more subtly than when reversing a sequence), and the ability to manipulate imagery tempo may only be available to movement experts. Smith and Wakefield (2013) further argue that as movement rhythms are crucial to success in sports, the rhythm of imagined actions should also receive more consideration, rather than recording only their overall timing. However, good paradigms to assess imagined movement rhythm need to be developed.

The temporal structure of auditory imagery has not received as much specific research attention, but given that rhythmic patterns are integral to music, the imagery of temporal structure is perhaps even more crucial to music imagination. Accordingly, musical experts were reported to show increased temporal acuity for imagery of tonal scales (Janata and Paroo, 2006), and temporal precision of musical imagery enhances music performance, especially in ensemble playing (Keller, 2012). However, even for non-experts, being able to imagine music unfolding in time and being able to track or scan its progression when hearing the music assumes the maintenance of an internal pulse or timekeeper (Povel and Essens, 1985) that dictates the pace of the (internal) music. As for movement, this process can be controlled, since, to a certain extent, we can imagine the same music at a very fast or slow tempo. However, after repeated listening, the tempo of music is stored in long-term memory, which was reported to be surprisingly accurate (Levitin and Cook, 1996). Thus, the timekeeper allows imagination processes to activate representations that unfold accurately over time. However, it is not yet clear how this timekeeper is implemented in the brain.

## The brain basis of imagined temporal patterns

Commonalities between the brain activity patterns of (auditory) rhythm perception and movement have long been interpreted as related to temporal structure (Ivry, 1996; Schubotz et al., 2000), implicating the cerebellum, striatum, premotor cortex (PMC), and (pre-) supplementary motor cortex (SMA). These areas are also part of a network of areas identified in a meta-analysis of 41 studies of interval timing tasks, where conjunctions for perceptual and motor timing tasks were reported in the bilateral SMA, PMC, parietal areas, bilateral inferior frontal gyrus (IFG), striatum, right insula and bilateral posterior cerebellum for sub-second intervals and in the bilateral SMA, left precentral gyrus, right cingulate gyrus, right IFG and bilateral insula for supra-second intervals (Wiener et al., 2010). Specifically for auditory perceptual timing, distinct patterns were found for duration- and beat-based timing processes (Teki et al., 2011), showing absolute, duration-based timing tasks to mostly activate cerebellar and brainstem areas, and relative, beat-based timing to activate the basal ganglia, PMC, (pre-)SMA and dorsolateral prefrontal cortex. Comparing these results to reports of imagined music and imagined movement reveals considerable anatomical overlap, with temporal processing being a common inference in the interpretation of these activations during imagination. Findings from music imagery provide consistent reports of activation in the secondary auditory cortex, IFG and (pre-)SMA, as well as cerebellar and striatal areas (Halpern and Zatorre, 1999; Leaver et al., 2009; Herholz et al., 2012). A recent meta-analysis of 75 movement imagery studies reported that when combining all types of movement, the areas that are most commonly found to be active during movement imagery include SMA, bilateral precentral gyrus, IFG, parietal areas, striatum, and cerebellum (lobule VI) (Hétu et al., 2013). Collectively, it appears that there is considerable overlap in the brain areas activated by music and motor imagery, and similar brain area networks are implicated in timing tasks. However, the striking apparent similarity between brain networks engaged in motor and perceptual timing, auditory imagination and imagined actions is of course no indication that each of these functions depends on identical neural mechanisms.

The investigation of temporal structure is inherently hampered by the fact that these structures are embedded in actions or percepts, which, as discussed above, are known to interact with imagery. However, this may be overcome by using ambiguous percepts or actions with different imposed temporal structures or interpretations, for instance by imposing different temporal structures on ambiguous stimuli (Fujioka et al., 2010; Schaefer et al., 2011a), or looking at individual differences in beat perception (Grahn and Mcauley, 2009). Using ambiguous stimuli to look at temporal structure, by imposing different temporal structures on identically performed actions, is much harder to achieve for actions. However, experiments looking directly at the common components of music and motor imagery can be easily conceived, for instance by directly comparing the manipulation of tempo in auditory and movement imagery. Thus, there are ways to overcome the complexity of the embedded, implicit nature of temporal patterns in experimental settings.

## Temporal skills and expertise

Imagery has long been thought of as an integral part of learning and memory, perception and action, information processing and reasoning (Kosslyn et al., 1995). Accurate (or veridical) imagery, be it of movements or music, is typically associated with expertise. Several investigations note increased vividness and/or accuracy for imagery of experts (Ozel et al., 2004; Janata and Paroo, 2006; Pecenka and Keller, 2009), and, in the case of musicians, accurate imagery is considered an integral aspect of music performance (Hargreaves, 2012) and a mark of good musicianship (as evident from historical musical aptitude scales; i.e., Gordon, 1965). Although this is correlational evidence, it may thus be the case that imagery skills are acquired with expertise. While for expert musicians strong temporal skills are clearly needed, the rhythm of movement is also highly important for effective sports performance (MacPherson et al., 2009), and again the implication is that temporal skills can be acquired. Temporal skills are not only important for music and movement, but are reported to be related to intelligence (Madison et al., 2009; Holm et al., 2011), and especially prospective timing may be related to attention and learning (Taatgen et al., 2007). Findings of increased executive functioning after music training (Moreno et al., 2011) could thus be interpreted as being related to increased temporal skills, potentially related to imagery ability. Although it is highly speculative at this point, one could hypothesize that the relation between cognitive control and imagery on one hand, the relation between imagery and temporal skills on the other, and the relation between temporal skills and cognitive ability, all depend on similar or overlapping neural mechanisms. This would have implications for the effects of training temporal skills for imagery ability (which has been identified as an issue in movement rehabilitation settings, cf. Malouin and Richards, 2010), as well as possibilities in cognitive training or rehabilitation.

In sum, I have argued that imagination of music and movement have shared components, which is supported by experimental evidence of their interactions and brain activations, and may be related to generating temporal patterns. Domain-specific expertise appears to be related to both imagery ability and temporal skills, with implications that are based on the known relations between both temporal skills and imagery skills and cognitive functioning. Future work, employing highly specific experimental designs, needs to clarify whether music and movement imagery timing are mediated by similar neural mechanisms, and whether increasing skills in one domain may carry over to increases in the other domain, or to cognitive ability.

### Conflict of interest statement

The author declares that the research was conducted in the absence of any commercial or financial relationships that could be construed as a potential conflict of interest.
